# CheckList for EvaluAtion of Radiomics research (CLEAR): a step-by-step reporting guideline for authors and reviewers endorsed by ESR and EuSoMII

**DOI:** 10.1186/s13244-023-01415-8

**Published:** 2023-05-04

**Authors:** Burak Kocak, Bettina Baessler, Spyridon Bakas, Renato Cuocolo, Andrey Fedorov, Lena Maier-Hein, Nathaniel Mercaldo, Henning Müller, Fanny Orlhac, Daniel Pinto dos Santos, Arnaldo Stanzione, Lorenzo Ugga, Alex Zwanenburg

**Affiliations:** 1 Department of Radiology, University of Health Sciences, Basaksehir Cam and Sakura City Hospital, Basaksehir, Istanbul, 34480 Turkey; 2 Institute of Diagnostic and Interventional Radiology, University Hospital Würzburg, Würzburg, Germany; 3 Center for Artificial Intelligence for Integrated Diagnostics (AI2D) & Center for Biomedical Image Computing & Analytics (CBICA), University of Pennsylvania, Philadelphia, PA USA; 4 Department of Radiology, Perelman School of Medicine, University of Pennsylvania, Philadelphia, PA USA; 5 Department of Pathology and Laboratory Medicine, Perelman School of Medicine, University of Pennsylvania, Philadelphia, PA USA; 6 Department of Medicine, Surgery, and Dentistry, University of Salerno, Baronissi, Italy; 7 Department of Radiology, Brigham and Women’s Hospital, Harvard Medical School, Boston, MA USA; 8 Division of Intelligent Medical Systems, German Cancer Research Center, Heidelberg, Germany; 9 National Center for Tumor Diseases (NCT), Heidelberg, Germany; 10 Institute for Technology Assessment, Massachusetts General Hospital, Boston, MA USA; 11 Department of Radiology, Massachusetts General Hospital, Boston, MA USA; 12 University of Applied Sciences of Western Switzerland (HES-SO Valais), Valais, Switzerland; 13 Department of Radiology and Medical Informatics, University of Geneva (UniGe), Geneva, Switzerland; 14 Laboratoire d’Imagerie Translationnelle en Oncologie (LITO)-U1288, Institut Curie, Inserm, Université PSL, Orsay, France; 15 Department of Radiology, University Hospital of Cologne, Cologne, Germany; 16 Institute for Diagnostic and Interventional Radiology, Goethe-University Frankfurt Am Main, Frankfurt, Germany; 17 Department of Advanced Biomedical Sciences, University of Naples “Federico II”, Naples, Italy; 18 OncoRay-National Center for Radiation Research in Oncology, Faculty of Medicine and University Hospital Carl Gustav Carus, Technische Universität Dresden, Helmholtz-Zentrum Dresden-Rossendorf, Dresden, Germany; 19 National Center for Tumor Diseases (NCT), Partner Site Dresden, Dresden, Germany; 20 German Cancer Research Center (DKFZ), Heidelberg, Germany

**Keywords:** Radiomics, Texture analysis, Checklist, Reporting, Imaging

## Abstract

Even though radiomics can hold great potential for supporting clinical decision-making, its current use is mostly limited to academic research, without applications in routine clinical practice. The workflow of radiomics is complex due to several methodological steps and nuances, which often leads to inadequate reporting and evaluation, and poor reproducibility. Available reporting guidelines and checklists for artificial intelligence and predictive modeling include relevant good practices, but they are not tailored to radiomic research. There is a clear need for a complete radiomics checklist for study planning, manuscript writing, and evaluation during the review process to facilitate the repeatability and reproducibility of studies. We here present a documentation standard for radiomic research that can guide authors and reviewers. Our motivation is to improve the quality and reliability and, in turn, the reproducibility of radiomic research. We name the checklist CLEAR (CheckList for EvaluAtion of Radiomics research), to convey the idea of being more transparent. With its 58 items, the CLEAR checklist should be considered a standardization tool providing the minimum requirements for presenting clinical radiomics research. In addition to a dynamic online version of the checklist, a public repository has also been set up to allow the radiomics community to comment on the checklist items and adapt the checklist for future versions. Prepared and revised by an international group of experts using a modified Delphi method, we hope the CLEAR checklist will serve well as a single and complete scientific documentation tool for authors and reviewers to improve the radiomics literature.

**Supplementary Information:**

The online version contains supplementary material available at 10.1186/s13244-023-01415-8.

## Key points

- The workflow of radiomics is complex with several methodological steps and nuances, which often leads to inadequate reproducibility, reporting, and evaluation.
- The CLEAR checklist proposes a single documentation standard for radiomics research that can guide authors, providing the minimum requirements for presenting clinical radiomics research.
- The CLEAR checklist aims to include all necessary items to support reviewer evaluation of radiomics-related manuscripts.

**Supplementary Information:**

The online version contains supplementary material available at 10.1186/s13244-023-01415-8.

## Introduction

Radiomics is the processing of medical images to use the resulting quantitative data for clinical decision-making [1]. A large number of articles have been published about radiomics in medical journals, with exponential growth in recent years [2, 3]. Even though radiomics could hold great potential for supporting clinical decision-making, its current use is mostly limited to academic research, with little to no impact on daily clinical practice [4, 5]. There are numerous reasons for this translational gap, such as challenges related to robustness, reproducibility, standardization, dataset design, choice of metrics, and code availability [6–8]. However, a major bottleneck toward understanding the roadblocks is the fact that the information on what exactly has been done in the radiomic studies is largely inaccessible due to poor reporting [9].

Only a few applications of published radiomic studies can be reproduced [10, 11]. The workflow of the radiomics pipeline is complex due to several steps and nuances, which may lead to inadequate reporting and thus the inability to reproduce findings [5, 12–14]. Unclear and incomplete reporting of study methodology and findings limit its critical appraisal, along with effective dissemination [15]. Authors of radiomic research publications must describe the methodology in adequate detail with supplementary data, code, and models to enable readers to reproduce the results [16].

Reporting checklists and guidelines have the potential to improve the quality of reporting and, in turn, the overall quality of research [17, 18]. Currently, there is no single checklist that focuses exclusively on radiomics covering all aspects of the research and evaluation process that are applicable to both authors and reviewers. There is an urgent need for rigorous reporting guidelines for radiomics to mature as a field [19–21]. The potential benefits of such a new checklist would be equally split between users (e.g., authors, researchers, and reviewers) and journals [22]. Users will be able to provide more reliable scientific information to the readers. Publishers will benefit from the improved trustworthiness of their journals by improving the rigor of radiomic publications.

Our purpose in this work is to develop a single documentation standard for radiomics research that can guide authors and reviewers. Our motivation is to improve the quality, reliability, and in turn, reproducibility of published radiomic research studies. To that end, we propose the CLEAR checklist, the CheckList for EvaluAtion of Radiomics research (Table 1), that should be included with each manuscript submission.

**Table 1:** CheckList for EvaluAtion of Radiomics research (CLEAR checklist)

Section	No	Item	Yes	No	n/a	Page
Title						
	1	Relevant title, specifying the radiomic methodology	☐	☐	☐	
Abstract						
	2	Structured summary with relevant information	☐	☐	☐	
Keywords						
	3	Relevant keywords for radiomics	☐	☐	☐	
Introduction						
	4	Scientific or clinical background	☐	☐	☐	
	5	Rationale for using a radiomic approach	☐	☐	☐	
	6	Study objective(s)	☐	☐	☐	
Method						
*Study Design*	7	Adherence to guidelines or checklists (e.g., CLEAR checklist)	☐	☐	☐	
	8	Ethical details (e.g., approval, consent, data protection)	☐	☐	☐	
	9	Sample size calculation	☐	☐	☐	
	10	Study nature (e.g., retrospective, prospective)	☐	☐	☐	
	11	Eligibility criteria	☐	☐	☐	
	12	Flowchart for technical pipeline	☐	☐	☐	
*Data*	13	Data source (e.g., private, public)	☐	☐	☐	
	14	Data overlap	☐	☐	☐	
	15	Data split methodology	☐	☐	☐	
	16	Imaging protocol (i.e., image acquisition and processing)	☐	☐	☐	
	17	Definition of non-radiomic predictor variables	☐	☐	☐	
	18	Definition of the reference standard (i.e., outcome variable)	☐	☐	☐	
*Segmentation*	19	Segmentation strategy	☐	☐	☐	
	20	Details of operators performing segmentation	☐	☐	☐	
*Pre-processing*	21	Image pre-processing details	☐	☐	☐	
	22	Resampling method and its parameters	☐	☐	☐	
	23	Discretization method and its parameters	☐	☐	☐	
	24	Image types (e.g., original, filtered, transformed)	☐	☐	☐	
*Feature extraction*	25	Feature extraction method	☐	☐	☐	
	26	Feature classes	☐	☐	☐	
	27	Number of features	☐	☐	☐	
	28	Default configuration statement for remaining parameters	☐	☐	☐	
*Data preparation*	29	Handling of missing data	☐	☐	☐	
	30	Details of class imbalance	☐	☐	☐	
	31	Details of segmentation reliability analysis	☐	☐	☐	
	32	Feature scaling details (e.g., normalization, standardization)	☐	☐	☐	
	33	Dimension reduction details	☐	☐	☐	
*Modeling*	34	Algorithm details	☐	☐	☐	
	35	Training and tuning details	☐	☐	☐	
	36	Handling of confounders	☐	☐	☐	
	37	Model selection strategy	☐	☐	☐	
*Evaluation*	38	Testing technique (e.g., internal, external)	☐	☐	☐	
	39	Performance metrics and rationale for choosing	☐	☐	☐	
	40	Uncertainty evaluation and measures (e.g., confidence intervals)	☐	☐	☐	
	41	Statistical performance comparison (e.g., DeLong's test)	☐	☐	☐	
	42	Comparison with non-radiomic and combined methods	☐	☐	☐	
	43	Interpretability and explainability methods	☐	☐	☐	
Results						
	44	Baseline demographic and clinical characteristics	☐	☐	☐	
	45	Flowchart for eligibility criteria	☐	☐	☐	
	46	Feature statistics (e.g., reproducibility, feature selection)	☐	☐	☐	
	47	Model performance evaluation	☐	☐	☐	
	48	Comparison with non-radiomic and combined approaches	☐	☐	☐	
Discussion						
	49	Overview of important findings	☐	☐	☐	
	50	Previous works with differences from the current study	☐	☐	☐	
	51	Practical implications	☐	☐	☐	
	52	Strengths and limitations (e.g., bias and generalizability issues)	☐	☐	☐	
Open Science						
*Data availability*	53	Sharing images along with segmentation data [n/e]	☐	☐	☐	
	54	Sharing radiomic feature data	☐	☐	☐	
*Code availability*	55	Sharing pre-processing scripts or settings	☐	☐	☐	
	56	Sharing source code for modeling	☐	☐	☐	
*Model availability*	57	Sharing final model files	☐	☐	☐	
	58	Sharing a ready-to-use system [n/e]	☐	☐	☐	

**Yes**, details provided; **No**, details not provided; **n/e**, not essential; **n/a**, not applicable

Note: Use the checklist in conjunction with the main text for clarification of all items. Fill the “Page” column with the related page number where the information is provided

## About CLEAR checklist

### Development

The checklist was designed by the lead author considering the previous efforts in the literature and subsequently revised by all other international co-authors with expertise in radiomics, deep learning, and statistics. A modified Delphi method was also utilized in the final selection of the items (see Additional file 1: S1 for all methodological details and results).

We name the checklist CLEAR (CheckList for EvaluAtion of Radiomics research), to convey the idea of being more transparent. It includes 58 items, providing the minimum requirements for presenting clinical radiomics research. Of these, 56 items are “essential” items. On the other hand, the remaining 2 (*Item#53* and *Item#58*) should be intended as “recommended” items.

A shortened version with 43 items was also presented as CLEAR-S (shortened version of CLEAR checklist) including only the methodological quality items that can be used for future systematic reviews.

### How to use

We advise using the checklist **(**Table 1**)** alongside the main text to ensure documentation of each checklist item. Additional file 2: S2 (without explanations), Additional file 3: S3 (with explanations), Additional file 4: S4 (CLEAR-S without explanations), and Additional file 5: S5 (CLEAR-S with explanations) allow users to download the checklists.

We strongly recommend using the online version of the checklist. It can easily be filled in and exported as PDF to submit as a supplement. The online version has a user-friendly design that prevents users from turning back to the main body of the paper for explanations of the items. The current version of the CLEAR checklist can be accessed with the following link (see https://clearchecklist.github.io/clear_checklist/CLEAR.html). The shortened version with 43 items (CLEAR-S) can also be accessed with the same link. Once the checklist is updated in the future, the same link will always provide the updated version and the older versions can be accessed via the repository of the CLEAR checklist (see https://github.com/clearchecklist/clear_checklist).

### How to contribute

We hope that the CLEAR checklist will stimulate discussion of the proposed items. We encourage the radiomics community to provide us with their views about how this checklist can be improved in future versions. To make the CLEAR checklist an online living or dynamic document, a public repository has been set up for the community to comment on and contribute to the checklist (see https://github.com/clearchecklist/clear_checklist).

Our policy for updating the guideline is as follows: *i,* evaluating community feedback; *ii,* group discussion for the update; *iii,* panelist votes; and *iv,* eventual update of guideline.

### Terminology used

This checklist uses the following terminology to be consistent. The terms “training set” and “validation set” are used for the data partitions with which the algorithm is trained and tuned, respectively. The term “test set” is used for the data with which the model is verified internally (i.e., with data from the same institution(s) as the training or validation sets) or externally (i.e., with independent data from different institution(s)). “Instance” is to indicate a single data element (e.g., lesion, tumor, or patient).

## Items of the CLEAR checklist

### Title

*Item#1. Relevant title, specifying the radiomic methodology.* Indicate the use of radiomics in the title. The following details can also be considered to be specified in the title: radiomic technique (e.g., hand-crafted, engineered, deep, delta, etc.), modality (e.g., computed tomography [CT], magnetic resonance imaging [MRI], ultrasound), important aspects of the scans (e.g., unenhanced, dynamic), use of machine learning (e.g., machine learning-based), external validation, and multi-center design.

### Abstract

*Item#2. Structured summary with relevant information.* Provide a structured summary of the purpose, methods, results, and conclusions, presenting only the most important aspects directly related to the purpose of the study. The abstract should be understandable on its own, without reading the main text. Considering the submission guidelines of the journals, it is recommended to specify the following items: the baseline characteristics (e.g., number of patients, scans, images, classes), data source (e.g., public, institutional), study nature (e.g., prospective, retrospective), segmentation technique (e.g., automated, semi-automated, or manual), feature extraction technique (e.g., hand-crafted, engineered, deep), dimensionality reduction techniques (e.g., feature selection, reproducibility analysis, multi-collinearity), modeling details (e.g., algorithms/models), validation technique (e.g., cross-validation), unseen testing (internal hold-out, external testing), model performance metrics (e.g., the area under the curve) with uncertainty measures (e.g., confidence intervals), number of the final set of features, traditional statistical methods with *p*-values, and open science status (e.g., public availability of data, code, and/or model).

### Keywords

*Item#3. Relevant keywords for radiomics.* List the primary keywords that indicate (e.g., radiomics, texture analysis) and characterize a radiomic study (e.g., machine learning, deep learning, computed tomography, magnetic resonance imaging, reproducibility), unless the journal requires exclusive use of certain terms (e.g., MeSH terms, which do not yet include radiomics-specific terms).

### Introduction section

*Item#4. Scientific or clinical background.* Define the scientific or clinical problem with a summary of the related literature and knowledge gaps, including a short review of the current state of knowledge. Describe why the scientific question is technically or clinically important.

*Item#5. Rationale for using a radiomic approach.* Describe why a radiomics approach is considered. Performance and problematic aspects of currently used methods need to be described. Mention what the radiomics approach would offer to solve these problems. Clearly state how radiomics could affect clinical practice considering the study question.

*Item#6. Study objective(s).* Describe the purpose of the study while focusing on the scientific problem. Mention the expected contributions to the current literature.

### Methods section

#### Study design

*Item#7. Adherence to guidelines or checklists (e.g., CLEAR checklist).* Indicate that the CLEAR checklist was used for reporting and submit the checklist as supplemental data. Do the same with other checklists or guidelines if used in addition to the CLEAR checklist.

*Item#8. Ethical details (e.g., approval, consent, data protection).* Describe the ethical questions to ensure that the study was conducted appropriately. Give information about ethical approval, informed consent, and data protection (e.g., de-identification) if the data are from private sources.

*Item#9. Sample size calculation.* Describe how the sample size or power was determined before or after the study (e.g., sample size/power calculation, based on availability).

*Item#10. Study nature (e.g., retrospective, prospective).* Indicate whether the study is prospective or retrospective and case/control or cohort, etc. In the case of prospective studies, provide registration details if available.

*Item#11. Eligibility criteria.* Define the inclusion criteria first. Then, specify the exclusion criteria. Avoid redundancies by using the opposite of the inclusion criteria as exclusion criteria. Specify the selection process (e.g., random, consecutive). Keep the numeric details of eligibility for the results.

*Item#12. Flowchart for technical pipeline.* Provide a flowchart for summarizing the key methodological steps in the study. Due to the complex nature of the radiomic approaches, such flowcharts help readers better understand the methodology.

#### Data

*Item#13. Data source (e.g., private, public).* State the data source (e.g., private, public, mixed [both private and public]). State clearly which data source is used in different data partitions. Provide web links and references if the source is public. Give the image or patient identifiers as a supplement if public data are used.

*Item#14. Data overlap.* State if any part of the dataset was used in a previous publication. Describe the differences between the current study and previous studies in terms of study purpose and methodology.

*Item#15. Data split methodology.* Describe the data split into training, validation, and test sets. Mention that multiple splits are created (e.g., k-fold cross-validation or bootstrapping). Specify how the assignment was done (e.g., random, semi-random, manual, center-wise, chronological order). Indicate the ratio of each partition, with class proportions. Describe at which level the data are split (e.g., patient-wise, image-wise, study-wise, scanner-wise, institution-wise). Clearly state the measures undertaken to avoid information leakage across datasets (e.g., creating the hold-out test set before feature normalization, feature selection, hyperparameter optimization, and model training) [23]. Note that any test data should only be used once for evaluation of the final model to prevent optimistic biases. Declare the systematic differences among the data partitions.

*Item#16. Imaging protocol (i.e., image acquisition and processing).* Provide the imaging protocol and acquisition parameters with post-processing details. Define physical pixel and voxel dimensions. Clearly state whether single or multiple or various scanners are used, with the number of instances for each protocol. Define the timing of the phase if a contrast medium was used. State the patient preparation (drug administration, blood sugar control before the scans, etc.) if performed.

*Item#17. Definition of non-radiomic predictor variables.* Describe the data elements appearing as non-radiomic predictors. Non-radiomic variables might be demographic characteristics (e.g., age, gender, ethnicity), widely used traditional laboratory biomarkers (e.g., carcinoembryonic antigen), or traditional approaches used in daily clinical practice (e.g., radiologist’s qualitative reading, Hounsfield Unit evaluation, Response Evaluation Criteria in Solid Tumors [RECIST], Response Assessment in Neuro-Oncology [RANO] criteria). It would be helpful to know how these predictors were identified (e.g., based on a literature review). If applicable, describe any transformation of predictors (e.g., binarization of continuous predictors, the grouping of levels of categorical variables).

*Item#18. Definition of the reference standard (i.e., outcome variable).* Describe the reference standard or outcome measure that the radiomic approach will predict (e.g., pathological grade, histopathological subtypes, genomic markers, local–regional control, survival, etc.). Provide the rationale for the choice of the reference standard (e.g., higher reproducibility rates). Clearly state the reproducibility concerns, potential biases, and limitations of the reference standard.

#### Segmentation

*Item#19. Segmentation strategy.* Indicate which software programs or tools are used for segmentation or annotation. Specify the version of the software and the exact configuration parameters. Provide reference and web link to the software. Describe the segmentation method (e.g., automatic, semi-automatic, manual). Provide the rules of the segmentation (e.g., margin shrinkage or expansion from the visible contour, included/excluded regions). Provide figures to show the segmentation style. Provide image registration details (e.g., software, version, link, parameters) if segmentation is propagated for multi-modal (e.g., CT and MR), multi-phase (e.g., unenhanced, arterial, venous phase CT), or multi-sequence (e.g., T2-weighted, post-contrast T1-weighted, diffusion-weighted imaging) analyses. If radiomic features are extracted from 2D images on a single slice, please explain with which criteria the slice is chosen. In the case of several lesions, explain if all the lesions are segmented and describe how the feature values are aggregated. If only one lesion is chosen, describe the criteria (e.g., the primitive or the most voluminous).

*Item#20 Details of operators performing segmentation.* State how many readers performed the segmentation, as well as their experience. In the case of multiple readers, describe how the final form of segmentation is achieved (e.g., the consensus of readers, intersection of segmentations, independent segmentation for further reproducibility analysis, sequential refinements from numerous expert raters until convergence), which is particularly important for the training data because the segmentation process on the test data should be as close to the clinical practice as possible, that is, the segmentation of a single reader.

#### Pre-processing

*Item#21. Image pre-processing details.* Indicate which software programs or tools are used for pre-processing. Specify the version of the software and the exact configuration parameters. Provide reference and web link to the software, if available. Describe all pre-processing techniques and associated parameters applied to the image including the normalization (e.g., minimum–maximum normalization, standardization, logarithmic transformation, bias field correction), de-noising, skull stripping (also known as brain extraction), interpolation to create uniform images (e.g., in terms of slice thickness), standardized uptake value conversion, and registration. Also, state if an image or feature-based harmonization technique was used.

*Item#22. Resampling method and its parameters.* Specify the resampling technique (e.g., linear, cubic b-spline) applied to the pixels or voxels. Provide the physical pixel and voxel dimensions after resampling.

*Item#23. Discretization method and its parameters.* Specify the discretization method (e.g., fixed bin width, fixed bin count method, or histogram equalization) used for hand-crafted radiomic feature extraction. Report the rationale for using a particular discretization technique. Indicate the number of gray levels for the fixed bin count method or the bin width as well as the value of the first level (or minimum and maximum bounds) for the fixed bin width method. Any experimental detail with different discretization methods and values is important to declare.

*Item#24. Image types (e.g., original, filtered, transformed).* Provide the image types from which the radiomic features are extracted, e.g., original or images with convolutional filters (e.g., Laplacian of Gaussian edge enhancement, wavelet decomposition) [24]. Also, give nuances about the parameters of transformed image types (e.g., sigma values of Laplacian of Gaussian filtering).

#### Feature extraction

*Item#25. Feature extraction method.* Indicate which software programs or tools are used for radiomic feature extraction. Specify the version of the software and the exact configuration parameters (also see *Item#55*). Provide reference and web link to the software. Indicate if the software adheres to the benchmarks/certification of IBSI [25]. Specify the general feature types, such as deep features, hand-crafted features, engineered features, or a combination. Refer to the mathematical formulas of the hand-crafted and engineered features. Provide formulas and code if new hand-crafted features are introduced. Present the architectural details for deep feature extraction. Provide details of any feature engineering performed. Specify whether radiomic features are extracted in a two-dimensional (2D) plane, 2D tri-planar, or three-dimensional (3D) space. If 2D features are extracted from 3D segmentation, provide reasons (e.g., large slice thickness) as to why such an approach is followed.

*Item#26. Feature classes.* Provide the radiomic feature classes (e.g., shape, first-order, gray-level co-occurrence matrix). Use IBSI terminology for feature classes [25]. Specify the number of features per feature class. Mention if any feature class is excluded with reason.

*Item#27. Number of features.* Indicate the total number of features per instance. If applicable, provide the number of features per imaging modality and its components (e.g., phase for CT, sequence for MRI, etc.).

*Item#28. Default configuration statement for remaining parameters.* After providing all modified parameters of pre-processing and radiomic feature extraction, state clearly that all other parameters remained as a default configuration.

#### Data preparation

*Item#29. Handling of missing data.* State if, and how much, missing data are present in the study. If so, provide details as to how it was addressed (e.g., deletion, substitution, or imputation).

*Item#30. Details of class imbalance.* Indicate the balance status of the classes according to the reference standard. Provide details about how class imbalance is handled. Specify the techniques (e.g., synthetic minority over-sampling, simple over-sampling through replication, under-sampling) used to achieve the class balance. Clearly state these data augmentation and under-sampling strategies are applied only in the training set.

*Item#31. Details of segmentation reliability analysis.* Describe the reliability analysis done to assess the influence of segmentation differences. An intra- and inter-rater reproducibility analysis must be considered in manual and semi-automatic methods. Provide details about the statistical tests used for the reliability analysis (e.g., intraclass correlation coefficient along with types) [26]. Mention the independence of assessment. Clearly state the reliability analysis is performed using the training set only.

*Item#32. Feature scaling details (e.g., normalization, standardization).* If applicable, describe the normalization technique applied to the radiomic feature data (e.g., minimum–maximum normalization, standardization, logarithmic transformation, ComBat normalization [choice of the batch, parametric or not, with or without empirical Bayes]). Specify the normalization scale. It is important to emphasize that this procedure is applied to the numeric radiomic feature data, not the images, in the training set and independently applied to the validation and test sets.

*Item#33. Dimension reduction details.* Specify the dimension reduction methods used, if applicable (e.g., collinearity analysis, reproducibility analysis, algorithm-based feature selection). Provide details about the statistical methods used. For example, provide the relevant statistical cut-off values for each step (e.g., features with intraclass correlation coefficient ≤ 0.9 are excluded). Clearly state the dimension reduction that is performed using the training set. Specify how the final number of features is achieved, for instance, the “rule of thumb” of ten features maximum for each instance.

#### Modeling

Of note, radiomics is not necessarily coupled with machine learning or traditional modeling. Conventional inferential statistics is also an option, particularly when the number of features is small.

*Item#34. Algorithm details.* Provide the name and version of software programs or packages used for modeling. Refer to the related publication of the software if available. Specify the algorithms used to create models with architectural details including inputs, outputs, and all intermediate components. The description of the architecture should be complete to allow for exact replication by other investigators (also see *Item#55 and Item#56*). When a previously described architecture is used, refer to the previous work and specify any modification. If the final model involved an ensemble of algorithms, specify the type of ensemble (e.g., stacking, majority voting, averaging, etc.).

*Item#35. Training and tuning details.* Describe the training process with adequate detail. Specify the augmentation technique, stopping criteria for training, hyperparameter tuning strategy (e.g., random, grid search, Bayesian), range of hyperparameter values used in tuning, optimization techniques, regularization parameters, and initialization of model parameters (e.g., random, transfer learning). If transfer learning is applied, clearly state which layers or parameters are frozen or affected.

*Item#36. Handling of confounders.* Describe the method (e.g., directed acyclic graphs) for the detection of potential confounders (e.g., differences in tumor size between cohorts, different image acquisition parameters such as slice thickness, and differences in patient populations between primary and secondary hospitals) [27, 28]. Please also describe how confounding was addressed (e.g., covariate adjustment).

*Item#37. Model selection strategy.* Describe how the final model was selected. Two broad categories for these are probabilistic (e.g., Akaike information criterion, Bayesian information criterion) and resampling methods (e.g., random train-test split, cross-validation, bootstrap validation) [12, 29]. Clearly state that only the training and validation sets are used for model selection. State if the model complexity is considered in selection, for instance, the “one standard error rule” [30]. Specify which performance metrics were used to select the final model.

#### Evaluation

*Item#38. Testing technique (e.g., internal, external).* Clearly state whether the model was internally or externally tested. The term “external testing” should only be used for the process that involves data usage from different institutions. In the case of external testing, specify the number of sites providing data and further details about whether they are used for multiple testing or in a single test. Describe the data characteristics and state if there are any differences among training, validation, internal testing, and external testing datasets (e.g., different scanners, different readers for segmentation, different ethnicity). Again, note that any test data should only be used once for evaluation to prevent biased performance metric estimates.

*Item#39. Performance metrics and rationale for choosing.* Specify the performance metrics to evaluate the predictive ability of the models. Justify the selected metrics according to the characteristics of the data (e.g., class imbalance). Beware of the potential pitfalls and follow recommendations when selecting the appropriate performance metrics [7, 31].

*Item#40. Uncertainty evaluation and measures (e.g., confidence intervals).* Describe the uncertainty evaluation (e.g., robustness, sensitivity analysis, calibration analysis if applicable) and measures of uncertainty quantification (e.g., confidence intervals, standard deviation).

*Item#41. Statistical performance comparison (e.g., DeLong's test).* Specify the statistical software and version used. Indicate which method was used for the comparison of the model performance such as the DeLong's test [32, 33], McNemar's test [34], or Bayesian approaches [35]. Provide a statistical threshold for the comparison (e.g., *p* < 0.05) along with confidence intervals if applicable to the method or metric. Also, state if multiplicity is considered and corrected when comparing multiple models (e.g., *p*-value adjustment, Bonferroni correction, false-discovery rate). Report threshold values to stratify data into groups for statistical testing (e.g., the operating point on the receiver operating characteristic [ROC] curve to define the confusion matrix, and cut-off values for defining strata in survival analysis).

*Item#42. Comparison with non-radiomic and combined methods.* Indicate whether comparisons with non-radiomic approaches (e.g., clinical parameters, laboratory parameters, traditional radiological evaluations) are performed. Non-radiomic approaches can be combined with radiomic data as well (e.g., clinical-radiomic evaluation). Explain how the clinical utility is assessed, such as with decision curve analysis [36].

*Item#43. Interpretability and explainability methods.* Describe the techniques used to increase the interpretability and explainability of the models created, if applicable [37]. Figures (e.g., class activation maps, feature maps, SHapley Additive exPlanations, accumulated local effects, partial dependence plots, etc.) related to the interpretability and explainability of the proposed radiomic model can be provided.

### Results section

*Item#44. Baseline demographic and clinical characteristics.* Provide the baseline demographic, clinical, and imaging characteristics in text and/or tables. Report the information separately for training, validation (i.e., cross-validation), and test datasets, along with grouping based on the reference standard or non-radiomic variables. Associated statistical tests should also be provided to identify if the sets are identical or not. Provide whether any confounder is detected and handled appropriately.

*Item#45. Flowchart for eligibility criteria.* Provide a flowchart for summarizing eligibility criteria with the number of included and excluded instances. If more than one data source is involved, please give details for each source separately.

*Item#46. Feature statistics (e.g., reproducibility, feature selection).* Give statistical information (e.g., distribution of features based on outcome variables) of the selected features for inclusion into the model. Provide the name and number of reproducible features (e.g., for segmentation reproducibility, for reproducibility against image perturbations). Create a table for the selected features with details of feature name, class, and image type. Also, provide results of reproducibility statistics. Reproducibility metrics of selected features can be presented in tables or supplementary files. Figures (e.g., boxplots, correlation matrix, feature importance plots) and tables of descriptive summaries of features can be provided.

*Item#47. Model performance evaluation.* Provide the performance metrics for training, validation (e.g., multiple splits like cross-validation, bootstrapping, etc.), and unseen test data, separately. A summary of the most important findings should be given in the text. Provide the ‘no information rate’ as well. Details can be provided in figures (e.g., ROC curves, precision–recall curves) and tables. It is a good practice to provide figures for calibration statistics to show the robustness of model performance. Present additional figures to showcase examples of true and false predictions to help readers better understand the strengths and limitations of the proposed strategy.

*Item#48. Comparison with non-radiomic and combined approaches.* Give the results about the comparison of radiomic approaches with non-radiomic (e.g., visual analysis, clinical only parameters) or combined approaches in the text and preferably on a table. Present the results for training, validation, and test data, separately. Provide uncertainty measures (e.g., confidence intervals, standard deviation, etc.) and statistical comparison results with *p*-values for each. Confusion matrices must also be provided. Aside from the predictive performance, specify which model is superior to others in terms of clinical utility. The clinical utility can be presented with a decision curve analysis. For the decision curve analysis, quantify the net benefit according to optimal probability thresholds, with multiple cut-points associated with different clinical views. Also, provide the rationale for why a specific threshold could be appropriate and clearly state what is meant by all and none strategies.

### Discussion section

*Item#49. Overview of important findings.* Provide a summary of the work and an overview of the most important findings. No statistical information is needed. Try to position the study into one of the following categories: proof of concept evaluation, technical task-specific evaluation, clinical evaluation, and post-deployment evaluation [38]. Summarize the contribution to the literature.

*Item#50. Previous works with differences from the current study.* Provide the most important and relevant previous works. Mention the most prominent differences between the current study and the previous works.

*Item#51. Practical implications.* Summarize the practical implications of the results. Describe the key impact of the work on the field. Highlight the potential clinical value and role of the study. Discuss any issues that may hamper the successful translation of the study into real-world clinical practice. Also, provide future expectations and possible next steps that others might build upon the current work.

*Item#52. Strengths and limitations (e.g., bias and generalizability issues).* Clearly state the strengths and the limitations of the current work. Any issue that may lead to potential bias, uncertainty, reproducibility, robustness, and generalizability problems should be declared.

### Open science

#### Data availability

*Item#53. Sharing images along with segmentation data.* (Please note that this item is “not essential” but “recommended.”) Provide relevant raw or processed image data considering the regulatory constraints of the institutions involved. Segmentation data can also be shared unless the segmentation is done as part of the workflow. In situations where sharing of the entire dataset is not possible, an end-to-end analysis workflow applied to a representative sample or a public dataset with similar characteristics can facilitate the ability of the readers in reproducing key components of the analysis [39]. Also, specify the reason if the data are not available.

*Item#54. Sharing radiomic feature data.* Share selected radiomic feature data along with clinical variables or labels with the public, if possible (i.e., in accordance with the regulatory constraints of the institute). Specify the reason if the radiomic feature data are not available.

#### Code availability

*Item#55. Sharing pre-processing scripts or settings.* Share the pre-processing and feature extraction parameter scripts or settings (e.g., YAML file in PyRadiomics or complete textual description). If it is not available in a script format, then the parameter configuration as appeared in the software program can be shared as a screenshot.

*Item#56. Sharing source code for modeling.* Share the modeling scripts [40]. Code scripts should include sufficient information to replicate the presented analysis (e.g., to train and test pipeline), with all dependencies and relevant comments to easily understand and build upon the method. Even if the actual input dataset used cannot be shared, in situations where a similar dataset is available publicly, it should be used to share an example workflow with all pre- and post-processing steps included. Specify the reason in case the source code is not available.

#### Model availability

*Item#57. Sharing final model files.* Share the final model files for internal or external testing [40]. Describe how inputs should be prepared to use the model. Also, include the source code that was used for pre-processing the input data. Specify the reason in case the final model data are not available.

*Item#58. Sharing a ready-to-use system.* (Please note this item is “not essential” but “recommended.”) An easy-to-use tool (e.g., standalone executable applications, notebooks, websites, virtual machines, etc.) can be created and shared with or without source code that is based on the model created [40]. The main aim is to be able to test or validate the model by other research groups. With this approach, users even without experience in machine learning or coding can also test the proposed models.

## Discussion

### Compliance with CLEAR checklist

Each CLEAR checklist item may not apply to all radiomic studies and their subsequent manuscripts, but all items should be considered. The items presented in the checklist should not be regarded as methodological recommendations but should be considered as reporting recommendations.

We strongly think compliance with items regarding the main structural elements of a manuscript, such as title, abstract, keywords, and introduction will help achieve improved visibility or more specifically discoverability and better attract the readers’ attention. Therefore, it facilitates the classification of a paper as relevant or irrelevant to the interests of the readers.

A radiomics workflow requires many choices, e.g., parameters for extracting radiomics features, and modeling. For instance, *Item#21* (Image pre-processing details), *Item#22* (Resampling method and its parameters), and Item*#23* (Discretization method and its parameters) are essential items to reproduce consistent and reproducible feature extraction, which are frequently underreported by the authors. Without systematic and complete reporting, it can become impossible to fully reproduce and externally validate a study. Therefore, compliance with the recommendations on the methods, results, and discussion sections will help achieve better and more transparent reporting and improve readers’ understanding of the findings.

We expect that compliance with the items related to open science will result in clarity in methodological steps and be a huge step for achieving complete transparency and reproducible research [40]. In this regard, *Item#54* (Sharing radiomic feature data)*, Item#55* (Sharing pre-processing scripts or settings)*, Item#56* (Sharing source code for modeling)*,* and *Item#57* (Sharing final model files) are essential open science items that are expected to be done by the authors. Reviewers particularly should check these and request if not provided. *Item#53* (Sharing images along with segmentation data) and *Item#58* (Sharing a ready-to-use system) are not essential but nonetheless highly recommended for full transparency of the study.

### Relevant previous checklists, guidelines, and quality scoring tools

Although not specifically designed for radiomic studies, a few manuscript checklists for artificial intelligence and statistical modeling have come into widespread use such as the Transparent Reporting of a multivariable prediction model for Individual Prognosis or Diagnosis (TRIPOD) and the CheckList for Artificial Intelligence in Medical Imaging (CLAIM) [41, 42]. Some new initiatives like Transparent Reporting of a multivariable prediction model for Individual Prognosis or Diagnosis-Artificial Intelligence (TRIPOD-AI) and Standards for Reporting of Diagnostic Accuracy Study-Artificial Intelligence (STARD-AI) are also on the way [43, 44]. These checklists include relevant good practices for radiomic studies. Nonetheless, not being tailored to radiomics and its specific technical details, they leave gaps concerning the needs of clinical radiomics research, manuscript writing, and evaluation during the review process. For example, CLAIM, being focused on artificial intelligence in medical imaging, lacks enough emphasis on the radiomic workflow and data sharing. The TRIPOD statement is broad and does not specifically deal with details important for radiomics such as feature extraction.

The Radiomics Quality Score (RQS) has been widely used to evaluate the methodological quality of radiomics research through systematic literature reviews [19]. However, some item definitions are not easy to interpret and require important expertise from the raters, leading to variable inter-reader reproducibility [45]. Furthermore, some RQS items may be too strict for most studies. For example, despite it being methodologically valuable in terms of robustness and clinical translation, it may not be possible to perform multiple scans and phantom studies in clinical practice, even in a prospective setting. Multiple scans or phantom studies are generally performed in isolation, not usually as part of a radiomic study with a clinical purpose. It should not be a requirement for every study. Establishing the robust radiomic features in multi-scan or phantom studies that are specifically designed for this purpose should suffice and guide future clinical studies. In addition, the Image Biomarker Standardization Initiative (IBSI) guidelines have been published, focusing more on documenting the computation process for individual features [10, 25]. Moreover, a recently published joint European Association of Nuclear Medicine/Society of Nuclear Medicine and Molecular Imaging (EANM/SNMMI) guideline on radiomics in nuclear medicine provides detailed information on best practices for both hand-crafted and deep learning-based radiomic approaches [46]. These guidelines can undeniably serve well to both authors and reviewers of radiomic publications but do not have the ease of use of a streamlined checklist. There is a radiomics-specific checklist that deserves to be mentioned here published by Pfaehler et al. [5]. However, this checklist was designed to cover only the reproducibility aspects of the radiomics studies, lacking modeling aspects and other advanced analytical tools.

### Strengths

We think our checklist has several key strengths that distinguish it from previous efforts. First, it provides complete coverage of radiomic research. Therefore, researchers do not need to apply or combine different checklists. For instance, one can use RQS for the methodological details [19], as it is mainly focused on handcrafted radiomics, and CLAIM for reporting the modeling components of radiomics research [42]. Additionally, the checklist of Pfaehler et al. can be used to assess the reproducibility aspects of radiomics [5]. However, CLEAR would be a viable alternative to comprehensively cover all parts of the study using a single checklist. Second, it was developed through a modified Delphi protocol with the involvement of 13 international experts. Third, the panel had enough diversity in terms of the multiple institutions involved. Fourth, we created a repository for the community to receive comments to improve the CLEAR checklist. This approach has the potential to fix any gaps in the checklist that become apparent during practical use. Fifth, we made the checklist a living or dynamic online document with a versioning system and a user-friendly design.

### Limitations

We have a few limitations to declare. First, the number of panelists is relatively low. It is recommended to have at least 10 individuals participate in a Delphi survey [47–49], which was achieved in each round. Second, the initial draft was designed by a single author, which might seem to lead to bias. Nonetheless, a sufficiently long period was provided to the panelists for their suggestions, comments, and revisions of the content in an online platform with discussion capabilities. This resulted in numerous discussions on several questions. We also performed a modified Delphi voting with a strict threshold. Furthermore, every significant issue raised in the final round was solved with additional quick voting. Third, the first author drafted the initial checklist which was refined by the expert panelists non-anonymously prior to the anonymous voting, which deviates from the anonymous principle of the standard process. Non-anonymous modified Delphi panels with in-person discussions are susceptible to a variety of process losses typical of group settings, including discussion dominance by one or a small number of participants and confirmation pressure, among others [50, 51]. However, research indicates that experts in panels that permit direct participant interaction are more likely to change their answers, reach a consensus, and demonstrate a deeper understanding of the reasons for disagreement than those in traditional Delphi panels [52]. Fourth, we only used a 3-point scale for the Delphi voting with an extra escape option. This was chosen because we did not intend to develop a scoring system. Fifth, although attempts were made to make the CLEAR comprehensive, it might not include all relevant items for reporting for all possible radiomic research questions. However, we hope our repository will be beneficial to bring these issues to the table for discussion and in turn potential consideration for the checklist. Sixth, the effectiveness and reproducibility of the CLEAR were not assessed, being outside the scope of this study. In the near future, we intend to evaluate these aspects in a dedicated research effort.

## Conclusions

The CLEAR checklist is a single and complete scientific documentation tool designed for authors and reviewers to improve the quality of designing and reporting clinical radiomics research. It provides a well-constructed framework for the key concepts to achieve high-quality and standardized scientific communication. Although some items may not apply to all radiomics studies, all items should be considered with care. We hope that the authors would benefit from this checklist when writing manuscripts and that all journals would adopt the CLEAR checklist for the peer review. We welcome comments, suggestions, and contributions to this guide in our repository to improve future versions of this checklist.

## Supplementary Information

**Additional file 1**. **Text S1**: Methods and results. **Figure S1**: Flowchart summarizing the key parts of the modified Delphi process in the development of CLEAR checklist. CLEAR, CheckList for EvaluAtion of Radiomics research.

**Additional file 2**. **Electronic Supplementary Material S2:** CLEAR checklist without explanations.

**Additional file 3**. **Electronic Supplementary Material S3**: CLEAR checklist with explanations.

**Additional file 4**. **Electronic Supplementary Material S4:** CLEAR-S checklist (shortened version) without explanations.

**Additional file 5**. **Electronic Supplementary Material S5:** CLEAR-S (shortened version) with explanations.

## Data Availability

Not applicable in this modified Delphi consensus paper.

## Abbreviations

2D: Two-dimensional
3D: Three-dimensional
CLAIM: CheckList for Artificial Intelligence in Medical imaging
CLEAR: CheckList for EvaluAtion of Radiomics research
CLEAR-S: Shortened version of CLEAR checklist
CT: Computed Tomography
IBSI: Image Biomarker Standardization Initiative
MRI: Magnetic Resonance Imaging
ROC: Receiver Operating Characteristic
RQS: Radiomics Quality Score
TRIPOD: Transparent Reporting of a multivariable prediction model for Individual Prognosis Or Diagnosis
