# Supplementary material for: CheckList for EvaluAtion of Radiomics research (CLEAR): a step-by-step reporting guideline for authors and reviewers endorsed by ESR and EuSoMII
Source: Insights Imaging. 2023 May 4;14:75. doi: 10.1186/s13244-023-01415-8 (PMC10160267; doi:10.1186/s13244-023-01415-8)
Supplement: Supplementary file 1 — Additional file 1. Text S1: Methods and results. Figure S1: Flowchart summarizing the key parts of the modified Delphi process in the development of CLEAR checklist. CLEAR, CheckList for EvaluAtion of Radiomics research. [file 13244_2023_1415_MOESM1_ESM.docx]

**Electronic Supplementary Material S1**

**Supplementary Text S1:** Methods and results.

**Methods**

***Design***

We conducted a maximum of three rounds of online modified Delphi surveys with international experts to determine which items on a prepared list could be modified, included, or excluded. Panelists evaluated and reached a consensus on the inclusion or exclusion of proposed items, in addition to suggesting additional items, independently, and anonymously (during voting). Following each round, participants received structured feedback on the previous round to reconcile individual perspectives and reach a group consensus. *Google Forms* was used to conduct the modified Delphi survey.

***Recruitment of participants***

Experts in radiomics, deep learning, or biostatistics from various countries were invited via an e-mail describing the development of the CLEAR checklist and explaining its purpose and procedure. Everyone who contributed to the development of the checklist at any stage was offered the opportunity to be a co-author of this publication. We intended to invite at least 10 individuals to participate in the modified Delphi survey [1–3]. In each round, the survey was open for a minimum of two weeks, and a reminder e-mail was sent one week, three days, and one day before the deadline. The deadlines were extended if fewer than 10 respondents attended the survey.

Before voting, participants' informed consent was obtained using an online form. Voting was anonymous, meaning that no one including the organizer knew the source of the answers provided by any participant. At any time, participants could withdraw from the study. Individuals who indicated a desire to decline the survey were planned to be removed from future invitations. Due to the anonymous nature of the voting, respondents could not retract their votes.

***Modified Delphi Procedure***

*Stage 1 (Preparation for modified Delphi exercise)*

A thorough literature review was conducted to identify potential checklist items. The lead author examined relevant papers, such as guidelines, checklists, systematic reviews, and narrative reviews, for the inclusion of potential items [4–14]. Invited participants collectively evaluated the items in an online platform with discussion. Participants were free to suggest adding, removing, merging, or modifying items. *Stage 1* was not anonymous.

Participants were requested to consider the following principles. First, the item must facilitate the reproducibility of a radiomic study. Second, the item should facilitate the evaluation of the quality and applicability of the radiomic study's results. Third, the list of items should represent the bare minimum of information that should be reported in all radiomic studies.

*Stage 2 (Modified Delphi exercise)*

Round 1 (first voting)

On a 3-point scale (Agree; Neutral; Disagree) with an escape option (No idea), participants were asked to rate the extent to which they agree with the inclusion of each item on the CLEAR checklist. Participants who agreed or were neutral regarding the inclusion of the item on the checklist were further asked for achieving agreement on the item's name and definition. Each item featured a free-text box for this purpose. In addition, a free-text box was provided at the end of the survey for participants to suggest additional checklist items. Before sending the survey to the panelists, usability and clarity were double-checked.

Round 2 (second voting)

The same participants were invited to participate in *Round 2* as in *Round 1*. Participants who were invited to participate in the first round, but did not respond, were also invited to participate in *Round 2*. Using the same format as *Round 1*, participants were presented with items for which there was no consensus, as well as any new items proposed during *Round 1*, and were asked to express their level of agreement with the inclusion of each item on the CLEAR checklist. Participants were given an anonymized group summary of the previous round on each item.

Round 3 (virtual discussion and consensus)

The purpose of *Round 3* was to discuss the results of the modified Delphi exercise and finalize the items to be included in the CLEAR checklist. It was conducted in virtual environments using group e-mail and the comment feature of Google Docs. All contributors from the preceding stages were invited. The discussion continued on both agreed and unresolved topics. Given the number of items and to streamline the checklist, any modification proposals were discussed and implemented by consensus. To resolve any major concern, new voting with a short deadline was performed when necessary.

***Statistical analysis***

After each of *Round 1* and *Round 2* was completed, the panel was e-mailed a summary of the item scores, including frequency and proportions across rating categories, along with comments. Any response with *no idea* was omitted from the agreement or disagreement statistics. The consensus was defined as either agreement (≥75% of participants agree) or disagreement (≥75% of participants disagree). It was described as no consensus if neither agreement nor disagreement was reached. The consensus items with the agreement in *Round 1* and *Round 2* were modified if there was any disagreement regarding the item's name and/or definition and then transferred to *Round 3* for virtual discussion and consensus meeting. The consensus items with disagreement were eliminated from the checklist without further discussion. The no consensus items were modified based on the comments provided in *Round 1* and *Round 2*, regardless of the status of agreement on the item's name and definition. Items with no consensus in *Round 1* and *Round 2* were voted for inclusion in the checklist in *Round 2* and *Round 3*, respectively. For any voting in *Round 3*, an absolute majority decision of all participants was sought for action on the issue.

**Results**

***Participants***

The organizer (lead author) invited 13 experts. Twelve of them accepted the invitation. The remaining 1 did not respond to the invitation. In total, 13 experts participated in at least one stage of the study. Four participants were from Germany, 3 from Italy, 3 from the United States, 1 from France, 1 from Switzerland, and 1 from Turkey.

***Stage 1 results***

Through literature review and experience, 76 items were prepared by the organizer, which was presented to the other participants in *Stage 1*. Ten experts were invited at this stage. In total, 11 experts including the organizer participated.

At the end of *Stage 1*, the number of items was reduced to 60, which also included new additions by other participants.

***Stage 2 results***

*Round 1*

Two new participants were invited to *Round 1*. In total, 11 experts completed *Round 1*. We prepared 60 items for *Round 1*. The agreement was achieved for 48 items for inclusion on the checklist. No consensus was achieved on 12 items. There was no disagreement.

According to the comments, 1 item with an agreement for inclusion on the checklist was split into 2 items, which resulted in 49 consensus items with an agreement.

*Round 2*

In total, 10 experts completed *Round 2*. Twelve items with no consensus in *Round 1* were presented to the experts in *Round 2*. An agreement was reached on 7 items for inclusion on the checklist. No consensus was achieved on 5 items. The no consensus items were as follows: *i*, cite the original source (for references section); *ii*, sharing image and segmentation data; *iii*, sharing a ready-to-use system; *iv*, competing interests; and *v*, supplementary files. There was no disagreement.

*Round 3*

At least 12 experts completed *Round 3*. Twelve experts participated in the open discussions (i.e., not anonymous). In this round, however, the precise number was unknown due to the use of additional anonymous polling.

From *Round 1* and *Round 2*, 49 (47 + 2 items produced by a split of one item) and 7 items with consensus for inclusion to the checklist were transferred to *Round 3*, respectively.

For the 5 items with no consensus from *Round 2*, we made an anonymous poll in *Round 3*, with two options as follows: *i*, removing all no consensus items, and *ii*, keeping 2 items about the data share (sharing image and segmentation data; sharing a ready-to-use system) and removing others. The absolute majority decided on keeping 2 items about the data share and removing the remaining items. We also made these two items (sharing image and segmentation data; sharing a ready-to-use system) non-essential in the checklist.

Due to a discussion raised among the participants, we made another anonymous voting in *Round 3* to further shorten the checklist only to the methodological and data sharing items. The absolute majority decided on the native version rather than the shortened one. Nevertheless, we also provided a shortened version (CLEAR-S) that may be useful for quality assessment in systematic reviews.

***Finalization of checklist***

In total, 58 items were included in the final CLEAR checklist, with the participation of 13 experts at least one stage of the study. **Supplementary Figure S1** summarizes the modified Delphi process in a flowchart.


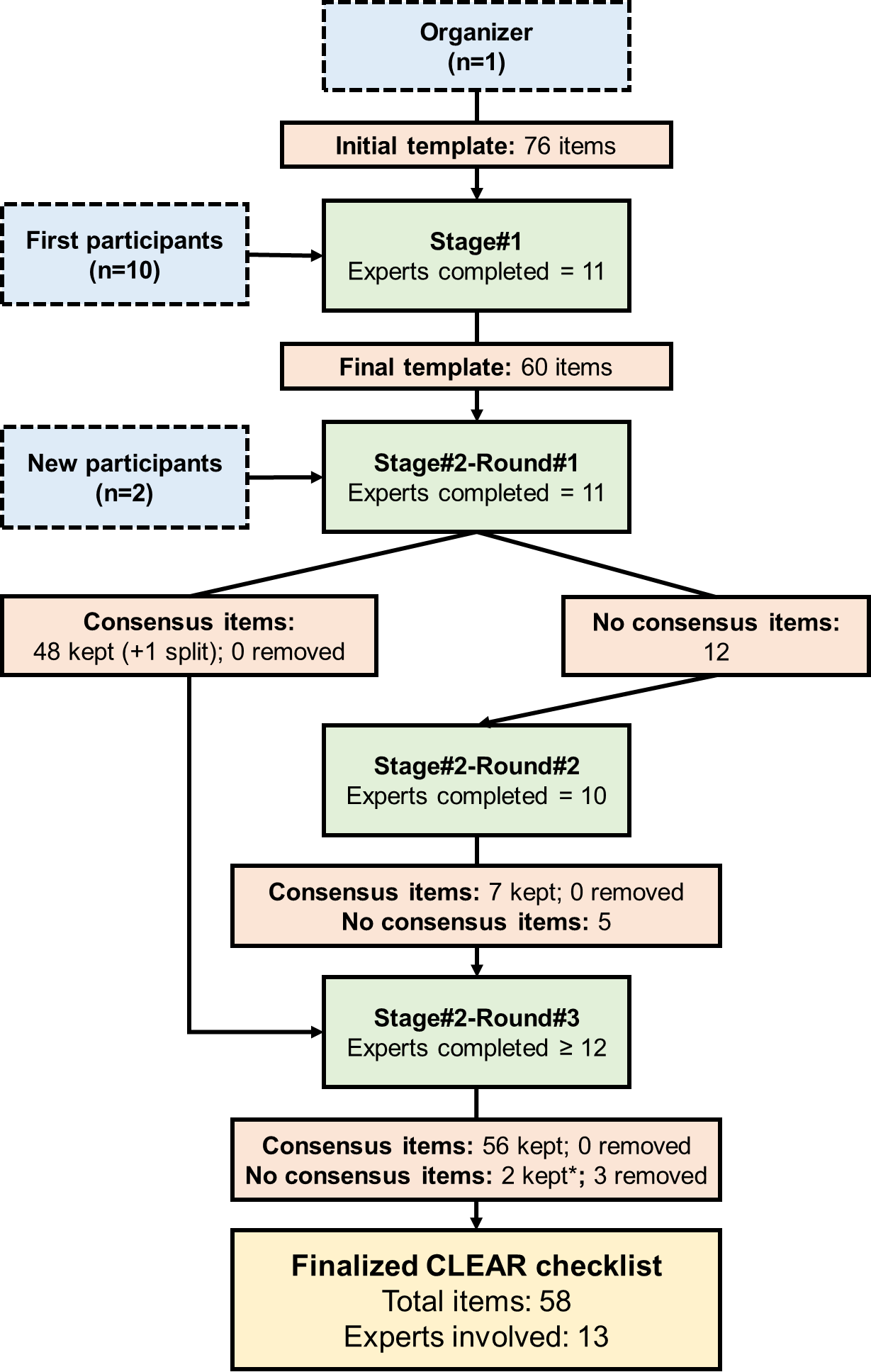


**Supplementary Figure S1:** Flowchart summarizing the key parts of the modified Delphi process in the development of the CLEAR checklist. *with additional voting in *Round 3*. ***CLEAR***, CheckList for EvaluAtion of Radiomics research

**References**

1. Veugelers R, Gaakeer MI, Patka P, Huijsman R (2020) Improving design choices in Delphi studies in medicine: the case of an exemplary physician multi-round panel study with 100% response. BMC Med Res Methodol 20:156. https://doi.org/10.1186/s12874-020-01029-4

2. Jünger S, Payne SA, Brine J, et al (2017) Guidance on Conducting and REporting DElphi Studies (CREDES) in palliative care: Recommendations based on a methodological systematic review. Palliat Med 31:684–706. https://doi.org/10.1177/0269216317690685

3. Paliwoda SJ (1983) Predicting the Future Using Delphi. Manag Decis 21:31–38. https://doi.org/10.1108/eb001309

4. Mongan J, Moy L, Kahn CE (2020) Checklist for Artificial Intelligence in Medical Imaging (CLAIM): A Guide for Authors and Reviewers. Radiol Artif Intell 2:e200029. https://doi.org/10.1148/ryai.2020200029

5. Kocak B, Durmaz ES, Ates E, Kilickesmez O (2019) Radiomics with artificial intelligence: a practical guide for beginners. Diagn Interv Radiol 25:485–495. https://doi.org/10.5152/dir.2019.19321

6. Vallières M, Zwanenburg A, Badic B, et al (2018) Responsible Radiomics Research for Faster Clinical Translation. J Nucl Med 59:189–193. https://doi.org/10.2967/jnumed.117.200501

7. Zwanenburg A, Vallières M, Abdalah MA, et al (2020) The Image Biomarker Standardization Initiative: Standardized Quantitative Radiomics for High-Throughput Image-based Phenotyping. Radiology 295:328–338. https://doi.org/10.1148/radiol.2020191145

8. Lambin P, Leijenaar RTH, Deist TM, et al (2017) Radiomics: the bridge between medical imaging and personalized medicine. Nat Rev Clin Oncol 14:749–762. https://doi.org/10.1038/nrclinonc.2017.141

9. Pfaehler E, Zhovannik I, Wei L, et al (2021) A systematic review and quality of reporting checklist for repeatability and reproducibility of radiomic features. Phys Imaging Radiat Oncol 20:69–75. https://doi.org/10.1016/j.phro.2021.10.007

10. Collins GS, Reitsma JB, Altman DG, Moons KGM (2015) Transparent reporting of a multivariable prediction model for individual prognosis or diagnosis (TRIPOD): the TRIPOD statement. BMJ 350:g7594. https://doi.org/10.1136/bmj.g7594

11. Norgeot B, Quer G, Beaulieu-Jones BK, et al (2020) Minimum information about clinical artificial intelligence modeling: the MI-CLAIM checklist. Nat Med 26:1320–1324. https://doi.org/10.1038/s41591-020-1041-y

12. Orlhac F, Nioche C, Klyuzhin I, et al (2021) Radiomics in PET Imaging. PET Clin 16:597–612. https://doi.org/10.1016/j.cpet.2021.06.007

13. Shur JD, Doran SJ, Kumar S, et al (2021) Radiomics in Oncology: A Practical Guide. RadioGraphics 41:1717–1732. https://doi.org/10.1148/rg.2021210037

14. van Timmeren JE, Cester D, Tanadini-Lang S, et al (2020) Radiomics in medical imaging—“how-to” guide and critical reflection. Insights Imaging 11:91. https://doi.org/10.1186/s13244-020-00887-2
