# Supplementary material for: CheckList for EvaluAtion of Radiomics research (CLEAR): a step-by-step reporting guideline for authors and reviewers endorsed by ESR and EuSoMII
Source: Insights Imaging. 2023 May 4;14:75. doi: 10.1186/s13244-023-01415-8 (PMC10160267; doi:10.1186/s13244-023-01415-8)
Supplement: Supplementary file 2 — Additional file 2. Electronic Supplementary Material S2: CLEAR checklist without explanations. [file 13244_2023_1415_MOESM2_ESM.docx]

**Electronic Supplementary Material S2:** CLEAR checklist without explanations

| Section | No. | Item | Yes | No | n/a | Page |
| --- | --- | --- | --- | --- | --- | --- |
| Title |  |  |  |  |  |  |
|  | 1 | Relevant title, specifying the radiomic methodology | ☐ | ☐ | ☐ |  |
| Abstract |  |  |  |  |  |  |
|  | 2 | Structured summary with relevant information | ☐ | ☐ | ☐ |  |
| Keywords |  |  |  |  |  |  |
|  | 3 | Relevant keywords for radiomics | ☐ | ☐ | ☐ |  |
| Introduction |  |  |  |  |  |  |
|  | 4 | Scientific or clinical background | ☐ | ☐ | ☐ |  |
|  | 5 | Rationale for using a radiomic approach | ☐ | ☐ | ☐ |  |
|  | 6 | Study objective(s) | ☐ | ☐ | ☐ |  |
| Method |  |  |  |  |  |  |
| *Study Design* | 7 | Adherence to guidelines or checklists (e.g., CLEAR checklist) | ☐ | ☐ | ☐ |  |
|  | 8 | Ethical details (e.g., approval, consent, data protection) | ☐ | ☐ | ☐ |  |
|  | 9 | Sample size calculation | ☐ | ☐ | ☐ |  |
|  | 10 | Study nature (e.g., retrospective, prospective) | ☐ | ☐ | ☐ |  |
|  | 11 | Eligibility criteria | ☐ | ☐ | ☐ |  |
|  | 12 | Flowchart for technical pipeline | ☐ | ☐ | ☐ |  |
| *Data* | 13 | Data source (e.g., private, public) | ☐ | ☐ | ☐ |  |
|  | 14 | Data overlap | ☐ | ☐ | ☐ |  |
|  | 15 | Data split methodology | ☐ | ☐ | ☐ |  |
|  | 16 | Imaging protocol (i.e., image acquisition and processing) | ☐ | ☐ | ☐ |  |
|  | 17 | Definition of non-radiomic predictor variables | ☐ | ☐ | ☐ |  |
|  | 18 | Definition of the reference standard (i.e., outcome variable) | ☐ | ☐ | ☐ |  |
| *Segmentation* | 19 | Segmentation strategy | ☐ | ☐ | ☐ |  |
|  | 20 | Details of operators performing segmentation | ☐ | ☐ | ☐ |  |
| *Pre-processing* | 21 | Image pre-processing details | ☐ | ☐ | ☐ |  |
|  | 22 | Resampling method and its parameters | ☐ | ☐ | ☐ |  |
|  | 23 | Discretization method and its parameters | ☐ | ☐ | ☐ |  |
|  | 24 | Image types (e.g., original, filtered, transformed) | ☐ | ☐ | ☐ |  |
| *Feature extraction* | 25 | Feature extraction method | ☐ | ☐ | ☐ |  |
|  | 26 | Feature classes | ☐ | ☐ | ☐ |  |
|  | 27 | Number of features | ☐ | ☐ | ☐ |  |
|  | 28 | Default configuration statement for remaining parameters | ☐ | ☐ | ☐ |  |
| *Data preparation* | 29 | Handling of missing data | ☐ | ☐ | ☐ |  |
|  | 30 | Details of class imbalance | ☐ | ☐ | ☐ |  |
|  | 31 | Details of segmentation reliability analysis | ☐ | ☐ | ☐ |  |
|  | 32 | Feature scaling details (e.g., normalization, standardization) | ☐ | ☐ | ☐ |  |
|  | 33 | Dimension reduction details | ☐ | ☐ | ☐ |  |
| *Modeling* | 34 | Algorithm details | ☐ | ☐ | ☐ |  |
|  | 35 | Training and tuning details | ☐ | ☐ | ☐ |  |
|  | 36 | Handling of confounders | ☐ | ☐ | ☐ |  |
|  | 37 | Model selection strategy | ☐ | ☐ | ☐ |  |
| *Evaluation* | 38 | Testing technique (e.g., internal, external) | ☐ | ☐ | ☐ |  |
|  | 39 | Performance metrics and rationale for choosing | ☐ | ☐ | ☐ |  |
|  | 40 | Uncertainty evaluation and measures (e.g., confidence intervals) | ☐ | ☐ | ☐ |  |
|  | 41 | Statistical performance comparison (e.g., DeLong’s test) | ☐ | ☐ | ☐ |  |
|  | 42 | Comparison with non-radiomic and combined methods | ☐ | ☐ | ☐ |  |
|  | 43 | Interpretability and explainability methods | ☐ | ☐ | ☐ |  |
| Results |  |  |  |  |  |  |
|  | 44 | Baseline demographic and clinical characteristics | ☐ | ☐ | ☐ |  |
|  | 45 | Flowchart for eligibility criteria | ☐ | ☐ | ☐ |  |
|  | 46 | Feature statistics (e.g., reproducibility, feature selection) | ☐ | ☐ | ☐ |  |
|  | 47 | Model performance evaluation | ☐ | ☐ | ☐ |  |
|  | 48 | Comparison with non-radiomic and combined approaches | ☐ | ☐ | ☐ |  |
| Discussion |  |  |  |  |  |  |
|  | 49 | Overview of important findings | ☐ | ☐ | ☐ |  |
|  | 50 | Previous works with differences from the current study | ☐ | ☐ | ☐ |  |
|  | 51 | Practical implications | ☐ | ☐ | ☐ |  |
|  | 52 | Strengths and limitations (e.g., bias and generalizability issues) | ☐ | ☐ | ☐ |  |
| Open Science |  |  |  |  |  |  |
| *Data availability* | 53 | Sharing images along with segmentation data [n/e] | ☐ | ☐ | ☐ |  |
|  | 54 | Sharing radiomic feature data | ☐ | ☐ | ☐ |  |
| *Code availability* | 55 | Sharing pre-processing scripts or settings | ☐ | ☐ | ☐ |  |
|  | 56 | Sharing source code for modeling | ☐ | ☐ | ☐ |  |
| *Model availability* | 57 | Sharing final model files | ☐ | ☐ | ☐ |  |
|  | 58 | Sharing a ready-to-use system [n/e] | ☐ | ☐ | ☐ |  |

**Yes**, details provided; **No**, details not provided; **n/e**, not essential; **n/a**, not applicable

Note: Use the checklist in conjunction with the main text for clarification of all items. Fill the “Page” column with the related page number where the information is provided.
