# Supplementary material for: CheckList for EvaluAtion of Radiomics research (CLEAR): a step-by-step reporting guideline for authors and reviewers endorsed by ESR and EuSoMII
Source: Insights Imaging. 2023 May 4;14:75. doi: 10.1186/s13244-023-01415-8 (PMC10160267; doi:10.1186/s13244-023-01415-8)
Supplement: Supplementary file 5 — Additional file 5. Electronic Supplementary Material S5: CLEAR-S (shortened version) with explanations. [file 13244_2023_1415_MOESM5_ESM.docx]

**Electronic Supplementary Material S5:** CLEAR-S (shortened version) with explanations

| Section | No. | Item | Yes | No | n/a | Page |
| --- | --- | --- | --- | --- | --- | --- |
| Method |  |  |  |  |  |  |
| *Study Design* | 7 | Adherence to guidelines or checklists (e.g., CLEAR checklist)  Indicate that the CLEAR checklist was used for reporting and submit the checklist as supplemental data. Do the same with other checklists or guidelines if used in addition to the CLEAR checklist. | ☐ | ☐ | ☐ |  |
|  | 8 | Ethical details (e.g., approval, consent, data protection)  Describe the ethical questions to ensure that the study was conducted appropriately. Give information about ethical approval, informed consent, and data protection (e.g., de-identification) if the data is from private sources. | ☐ | ☐ | ☐ |  |
|  | 9 | Sample size calculation  Describe how the sample size or power was determined before or after the study (e.g., sample size/power calculation, based on availability). | ☐ | ☐ | ☐ |  |
|  | 10 | Study nature (e.g., retrospective, prospective)  Indicate whether the study is prospective or retrospective and case/control or cohort, etc. In the case of prospective studies, provide registration details if available. | ☐ | ☐ | ☐ |  |
|  | 11 | Eligibility criteria  Define the inclusion criteria first. Then, specify the exclusion criteria. Avoid redundancies by using the opposite of the inclusion criteria as exclusion criteria. Specify the selection process (e.g., random, consecutive). Keep the numeric details of eligibility for the results. | ☐ | ☐ | ☐ |  |
|  | 12 | Flowchart for technical pipeline  Provide a flowchart for summarizing the key methodological steps in the study. Due to the complex nature of the radiomic approaches, such flowcharts help readers better understand the methodology. | ☐ | ☐ | ☐ |  |
| *Data* | 13 | Data source (e.g., private, public)  State the data source (e.g., private, public, mixed [both private and public]). State clearly which data source is used in different data partitions. Provide web links and references if the source is public. Give the image or patient identifiers as a supplement if public data is used. | ☐ | ☐ | ☐ |  |
|  | 14 | Data overlap  State if any part of the dataset was used in a previous publication. Describe the differences between the current study and previous studies in terms of study purpose and methodology. | ☐ | ☐ | ☐ |  |
|  | 15 | Data split methodology  Describe the data split into training, validation, and test sets. Mention that multiple splits are created (e.g., k-fold cross-validation or bootstrapping). Specify how the assignment was done (e.g., random, semi-random, manual, center-wise, chronological order). Indicate the ratio of each partition, with class proportions. Describe at which level the data is split (e.g., patient-wise, image-wise, study-wise, scanner-wise, institution-wise). Clearly state the measures undertaken to avoid information leakage across datasets (e.g., creating the hold-out test set before feature normalization, feature selection, hyperparameter optimization, and model training) [23]. Note that any test data should only be used once for evaluation of the final model to prevent optimistic biases. Declare the systematic differences among the data partitions. | ☐ | ☐ | ☐ |  |
|  | 16 | Imaging protocol (i.e., image acquisition and processing)  Provide the imaging protocol and acquisition parameters with post-processing details. Define physical pixel and voxel dimensions. Clearly state whether single or multiple or various scanners are used, with the number of instances for each protocol. Define the timing of the phase if a contrast medium was used. State the patient preparation (drug administration, blood sugar control before the scans, etc.) if performed. | ☐ | ☐ | ☐ |  |
|  | 17 | Definition of non-radiomic predictor variables  Describe the data elements appearing as non-radiomic predictors. Non-radiomic variables might be demographic characteristics (e.g., age, gender, ethnicity), widely used traditional laboratory biomarkers (e.g., carcinoembryonic antigen), or traditional approaches used in daily clinical practice (e.g., radiologist’s qualitative reading, Hounsfield Unit evaluation, Response Evaluation Criteria in Solid Tumors (RECIST), Response Assessment in Neuro-Oncology (RANO) criteria). It would be helpful to know how these predictors were identified (e.g., based on a literature review). If applicable, describe any transformation of predictors (e.g., binarization of continuous predictors, the grouping of levels of categorical variables). | ☐ | ☐ | ☐ |  |
|  | 18 | Definition of the reference standard (i.e., outcome variable)  Describe the reference standard or outcome measure that the radiomic approach will predict (e.g., pathological grade, histopathological subtypes, genomic markers, local-regional control, survival, etc.). Provide the rationale for the choice of the reference standard (e.g., higher reproducibility rates). Clearly state the reproducibility concerns, potential biases, and limitations of the reference standard. | ☐ | ☐ | ☐ |  |
| *Segmentation* | 19 | Segmentation strategy  Indicate which software programs or tools are used for segmentation or annotation. Specify the version of the software and the exact configuration parameters. Provide reference and web link to the software. Describe the segmentation method (e.g., automatic, semi-automatic, manual). Provide the rules of the segmentation (e.g., margin shrinkage or expansion from the visible contour, included/excluded regions). Provide figures to show the segmentation style. Provide image registration details (e.g., software, version, link, parameters) if segmentation is propagated for multi-modal (e.g., CT and MR), multi-phase (e.g., unenhanced, arterial, venous phase CT), or multi-sequence (e.g., T2-weighted, post-contrast T1-weighted, diffusion-weighted imaging) analyses. If radiomic features are extracted from 2D images on a single slice, please explain with which criteria the slice is chosen. In the case of several lesions, explain if all the lesions are segmented and describe how the feature values are aggregated. If only one lesion is chosen, describe the criteria (e.g., the primitive or the most voluminous). | ☐ | ☐ | ☐ |  |
|  | 20 | Details of operators performing segmentation  State how many readers performed the segmentation, as well as their experience. In the case of multiple readers, describe how the final form of segmentation is achieved (e.g., the consensus of readers, intersection of segmentations, independent segmentation for further reproducibility analysis, sequential refinements from numerous expert raters until convergence), which is particularly important for the training data because the segmentation process on the test data should be as close to the clinical practice as possible, that is, the segmentation of a single reader. | ☐ | ☐ | ☐ |  |
| *Pre-processing* | 21 | Image pre-processing details  Indicate which software programs or tools are used for pre-processing. Specify the version of the software and the exact configuration parameters. Provide reference and web link to the software, if available. Describe all pre-processing techniques and associated parameters applied to the image including the normalization (e.g., minimum-maximum normalization, standardization, logarithmic transformation, bias field correction), de-noising, skull stripping (also known as brain extraction), interpolation to create uniform images (e.g., in terms of slice thickness), standardized uptake value conversion, and registration. Also, state if an image or feature-based harmonization technique was used. | ☐ | ☐ | ☐ |  |
|  | 22 | Resampling method and its parameters  Specify the resampling technique (e.g., linear, cubic b-spline) applied to the pixels or voxels. Provide the physical pixel and voxel dimensions after resampling. | ☐ | ☐ | ☐ |  |
|  | 23 | Discretization method and its parameters  Specify the discretization method (e.g., fixed bin width, fixed bin count method, or histogram equalization) used for hand-crafted radiomic feature extraction. Report the rationale for using a particular discretization technique. Indicate the number of grey levels for the fixed bin count method or the bin width as well as the value of the first level (or minimum and maximum bounds) for the fixed bin width method. Any experimental detail with different discretization methods and values is important to declare. | ☐ | ☐ | ☐ |  |
|  | 24 | Image types (e.g., original, filtered, transformed)  Provide the image types from which the radiomic features are extracted, e.g., original or images with convolutional filters (e.g., Laplacian of Gaussian edge enhancement, wavelet decomposition) [24]. Also, give nuances about the parameters of transformed image types (e.g., sigma values of Laplacian of Gaussian filtering). | ☐ | ☐ | ☐ |  |
| *Feature extraction* | 25 | Feature extraction method  Indicate which software programs or tools are used for radiomic feature extraction. Specify the version of the software and the exact configuration parameters (also see Item#55). Provide reference and web link to the software. Indicate if the software adheres to the benchmarks/certification of IBSI [25]. Specify the general feature types, such as deep features, hand-crafted features, engineered features, or a combination. Refer to the mathematical formulas of the hand-crafted and engineered features. Provide formulas and code if new hand-crafted features are introduced. Present the architectural details for deep feature extraction. Provide details of any feature engineering performed. Specify whether radiomic features are extracted in a two-dimensional (2D) plane, 2D tri-planar, or three-dimensional (3D) space. If 2D features are extracted from 3D segmentation, provide reasons (e.g., large slice thickness) as to why such an approach is followed. | ☐ | ☐ | ☐ |  |
|  | 26 | Feature classes  Provide the radiomic feature classes (e.g., shape, first-order, grey-level co-occurrence matrix). Use IBSI terminology for feature classes [25]. Specify the number of features per feature class. Mention if any feature class is excluded with reason. | ☐ | ☐ | ☐ |  |
|  | 27 | Number of features  Indicate the total number of features per instance. If applicable, provide the number of features per imaging modality and its components (e.g., phase for CT, sequence for MRI, etc.). | ☐ | ☐ | ☐ |  |
|  | 28 | Default configuration statement for remaining parameters  After providing all modified parameters of pre-processing and radiomic feature extraction, state clearly that all other parameters remained as a default configuration. | ☐ | ☐ | ☐ |  |
| *Data preparation* | 29 | Handling of missing data  State if, and how much, missing data are present in the study. If so, provide details as to how it was addressed (e.g., deletion, substitution, or imputation). | ☐ | ☐ | ☐ |  |
|  | 30 | Details of class imbalance  Indicate the balance status of the classes according to the reference standard. Provide details about how class imbalance is handled. Specify the techniques (e.g., synthetic minority over-sampling, simple over-sampling through replication, under-sampling) used to achieve the class balance. Clearly state these data augmentation and under-sampling strategies are applied only in the training set. | ☐ | ☐ | ☐ |  |
|  | 31 | Details of segmentation reliability analysis  Describe the reliability analysis done to assess the influence of segmentation differences. An intra- and inter-rater reproducibility analysis must be considered in manual and semi-automatic methods. Provide details about the statistical tests used for the reliability analysis (e.g., intraclass correlation coefficient along with types) [26]. Mention the independence of assessment. Clearly state the reliability analysis is performed using the training set only. | ☐ | ☐ | ☐ |  |
|  | 32 | Feature scaling details (e.g., normalization, standardization)  If applicable, describe the normalization technique applied to the radiomic feature data (e.g., minimum-maximum normalization, standardization, logarithmic transformation, ComBat normalization [choice of the batch, parametric or not, with or without empirical Bayes]). Specify the normalization scale. It is important to emphasize that this procedure is applied to the numeric radiomic feature data, not the images, in the training set and independently applied to the validation and test sets. | ☐ | ☐ | ☐ |  |
|  | 33 | Dimension reduction details  Specify the dimension reduction methods used, if applicable (e.g., collinearity analysis, reproducibility analysis, algorithm-based feature selection). Provide details about the statistical methods used. For example, provide the relevant statistical cut-off values for each step (e.g., features with intraclass correlation coefficient ≤0.9 are excluded). Clearly state the dimension reduction that is performed using the training set. Specify how the final number of features is achieved, for instance, the “rule of thumb” of ten features maximum for each instance. | ☐ | ☐ | ☐ |  |
| *Modeling* | 34 | Algorithm details  Provide the name and version of software programs or packages used for modeling. Refer to the related publication of the software if available. Specify the algorithms used to create models with architectural details including inputs, outputs, and all intermediate components. The description of the architecture should be complete to allow for exact replication by other investigators (also see Item#55 and Item#56). When a previously described architecture is used, refer to the previous work and specify any modification. If the final model involved an ensemble of algorithms, specify the type of ensemble (e.g., stacking, majority voting, averaging, etc.). | ☐ | ☐ | ☐ |  |
|  | 35 | Training and tuning details  Describe the training process with adequate detail. Specify the augmentation technique, stopping criteria for training, hyperparameter tuning strategy (e.g., random, grid-search, Bayesian), range of hyperparameter values used in tuning, optimization techniques, regularization parameters, and initialization of model parameters (e.g., random, transfer learning). If transfer learning is applied, clearly state which layers or parameters are frozen or affected. | ☐ | ☐ | ☐ |  |
|  | 36 | Handling of confounders  Describe the method (e.g., directed acyclic graphs) for the detection of potential confounders (e.g., differences in tumor size between cohorts, different image acquisition parameters such as slice thickness, and differences in patient populations between primary and secondary hospitals) [27, 28]. Please describe how confounding was addressed (e.g., covariate adjustment). | ☐ | ☐ | ☐ |  |
|  | 37 | Model selection strategy  Describe how the final model was selected. Two broad categories for these are probabilistic (e.g., Akaike information criterion, Bayesian information criterion) and resampling methods (e.g., random train-test split, cross-validation, bootstrap validation) [12, 29]. Clearly state that only the training and validation sets are used for model selection. State if the model complexity is considered in selection, for instance, the “one standard error rule” [30]. Specify which performance metrics were used to select the final model. | ☐ | ☐ | ☐ |  |
| *Evaluation* | 38 | Testing technique (e.g., internal, external)  Clearly state whether the model was internally or externally tested. The term “external testing” should only be used for the process that involves data usage from different institutions. In the case of external testing, specify the number of sites providing data and further details about whether they are used for multiple testing or in a single test. Describe the data characteristics and state if there are any differences among training, validation, internal testing, and external testing datasets (e.g., different scanners, different readers for segmentation, different ethnicity). Again, note that any test data should only be used once for evaluation to prevent biased performance metric estimates. | ☐ | ☐ | ☐ |  |
|  | 39 | Performance metrics and rationale for choosing  Specify the performance metrics to evaluate the predictive ability of the models. Justify the selected metrics according to the characteristics of the data (e.g., class imbalance). Beware of the potential pitfalls and follow recommendations when selecting the appropriate performance metrics [7, 31]. | ☐ | ☐ | ☐ |  |
|  | 40 | Uncertainty evaluation and measures (e.g., confidence intervals)  Describe the uncertainty evaluation (e.g., robustness, sensitivity analysis, calibration analysis if applicable) and measures of uncertainty quantification (e.g., confidence intervals, standard deviation). | ☐ | ☐ | ☐ |  |
|  | 41 | Statistical performance comparison (e.g., DeLong’s test)  Specify the statistical software and version used. Indicate which method was used for the comparison of the model performance such as the DeLong’s test [32, 33], McNemar’s test [34], or Bayesian approaches [35]. Provide a statistical threshold for the comparison (e.g., p<0.05) along with confidence intervals if applicable to the method or metric. Also, state if multiplicity is considered and corrected when comparing multiple models (e.g., p-value adjustment, Bonferroni correction, false-discovery rate). Report threshold values to stratify data into groups for statistical testing (e.g., the operating point on the receiver operating characteristic [ROC] curve to define the confusion matrix, and cut-off values for defining strata in survival analysis). | ☐ | ☐ | ☐ |  |
|  | 42 | Comparison with non-radiomic and combined methods  Indicate whether comparisons with non-radiomic approaches (e.g., clinical parameters, laboratory parameters, traditional radiological evaluations) are performed. Non-radiomic approaches can be combined with radiomic data as well (e.g., clinical-radiomic evaluation). Explain how the clinical utility is assessed, such as with decision curve analysis [36]. | ☐ | ☐ | ☐ |  |
|  | 43 | Interpretability and explainability methods  Describe the techniques used to increase the interpretability and explainability of the models created, if applicable [37]. Figures (e.g., class activation maps, feature maps, SHapley Additive exPlanations, accumulated local effects, partial dependence plots, etc.) related to the interpretability and explainability of the proposed radiomic model can be provided. | ☐ | ☐ | ☐ |  |
| Open Science |  |  |  |  |  |  |
| *Data availability* | 53 | Sharing images along with segmentation data  [Please note this item is “not essential” but “recommended”] Provide relevant raw or processed image data considering the regulatory constraints of the institutions involved. Segmentation data can also be shared unless the segmentation is done as part of the workflow. In situations where sharing of the entire dataset is not possible, an end-to-end analysis workflow applied to a representative sample, or a public dataset with similar characteristics can facilitate the ability of the readers in reproducing key components of the analysis [39]. Also, specify the reason if the data is not available. | ☐ | ☐ | ☐ |  |
|  | 54 | Sharing radiomic feature data  Share selected radiomic feature data along with clinical variables or labels with the public, if possible (i.e., in accordance with the regulatory constraints of the institute). Specify the reason if the radiomic feature data is not available. | ☐ | ☐ | ☐ |  |
| *Code availability* | 55 | Sharing pre-processing scripts or settings  Share the pre-processing and feature extraction parameter scripts or settings (e.g., YAML file in PyRadiomics or complete textual description). If it is not available in a script format, then the parameter configuration as appeared in the software program can be shared as a screenshot. | ☐ | ☐ | ☐ |  |
|  | 56 | Sharing source code for modeling  Share the modeling scripts [40]. Code scripts should include sufficient information to replicate the presented analysis (e.g., to train and test pipeline), with all dependencies and relevant comments to easily understand and build upon the method. Even if the actual input dataset used cannot be shared, in situations where a similar dataset is available publicly, it should be used to share an example workflow with all pre- and post-processing steps included. Specify the reason in case the source code is not available. | ☐ | ☐ | ☐ |  |
| *Model availability* | 57 | Sharing final model files  Share the final model files for internal or external testing [40]. Describe how inputs should be prepared to use the model. Also, include the source code that was used for pre-processing the input data. Specify the reason in case the final model data is not available. | ☐ | ☐ | ☐ |  |
|  | 58 | Sharing a ready-to-use system  [Please note this item is “not essential” but “recommended”] An easy-to-use tool (e.g., standalone executable applications, notebooks, websites, virtual machines, etc.) can be created and shared with or without source code that is based on the model created [40]. The main aim is to be able to test or validate the model by other research groups. With this approach, users even without experience in machine learning or coding can also test the proposed models. | ☐ | ☐ | ☐ |  |

**Yes**, details provided; **No**, details not provided; **n/a**, not applicable

Note: Use the checklist in conjunction with the main text for clarification of all items. Fill the “Page” column with the related page number where the information is provided.
